# Cutaneous involvement in multiple myeloma. A rare entity

**DOI:** 10.1002/ccr3.9476

**Published:** 2025-01-24

**Authors:** L. Rapparini, A. Pileri, S. Robuffo, C. Agostinelli, Michelangelo La Placa

**Affiliations:** ^1^ Dermatology Unit IRCCS Azienda Ospedaliero‐Universitaria di Bologna Bologna Italy; ^2^ Department of Medical and Surgical Sciences Alma Mater Studiorum University of Bologna Italy; ^3^ Haematopathology Unit IRCCS Azienda Ospedaliero‐Universitaria di Bologna Bologna Italy

**Keywords:** cutaneous, metastasis, multiple myeloma, secondarism

## Abstract

Cutaneous involvement in multiple myeloma is rare and may present as nodules mimicking other lymphoid neoplasms. It typically occurs late in the course of the disease and is associated with an aggressive clinical course and poor prognosis.

## Graphical abstract

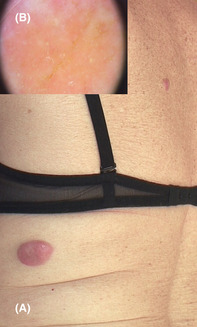

## 1 CASE HISTORY

A 74‐year‐old woman presented to our outpatient service with two pinkish nodules on the dorsa, which had been present for 5 months. She was undergoing treatment with elotuzumab‐pomalidomide‐dexamethasone for IgG/kappa multiple myeloma, which had developed 4 years earlier from monoclonal gammopathy. Her medical history included a left nephroadrenectomy for renal angiomyolipoma, surgical treatment for high‐grade papillary urothelial carcinoma of the bladder and papillary carcinoma of the right lobe of the thyroid, both of which were in apparent remission. Additionally, she had a history of hypercalcemia, anemia, and chronic renal disease.

**FIGURE 1:**
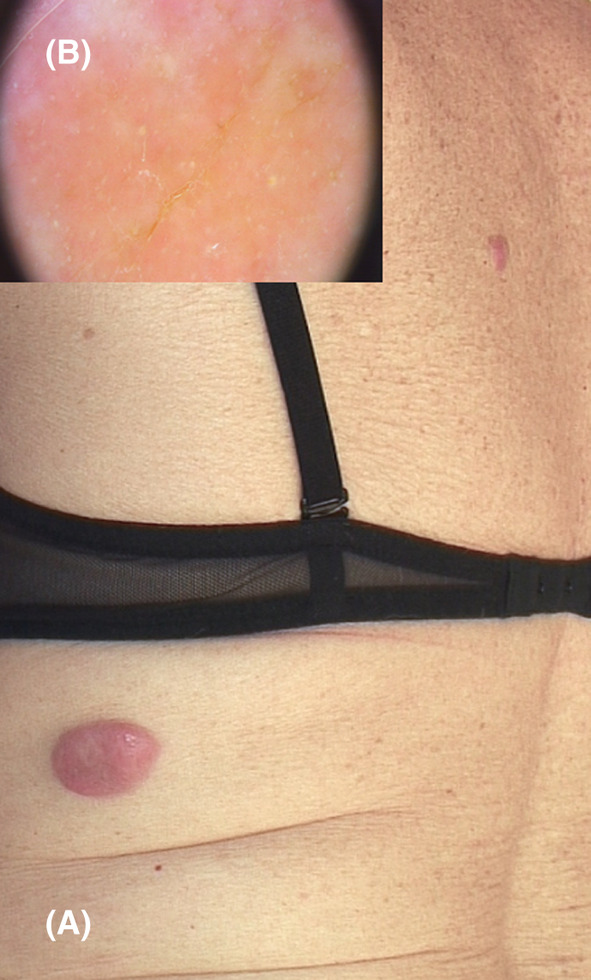
(A) Pink‐reddish nodules on the back. (B) Dermoscopy of the larger nodule showing superficial scales overlying a yellowish background.

During our consultation, we observed a 6‐cm infiltrated nodule on her lower back and a smaller nodule on her upper back, both exhibiting a pink‐reddish appearance (Figure 1A).

## 2 INVESTIGATIONS

**FIGURE 2:**
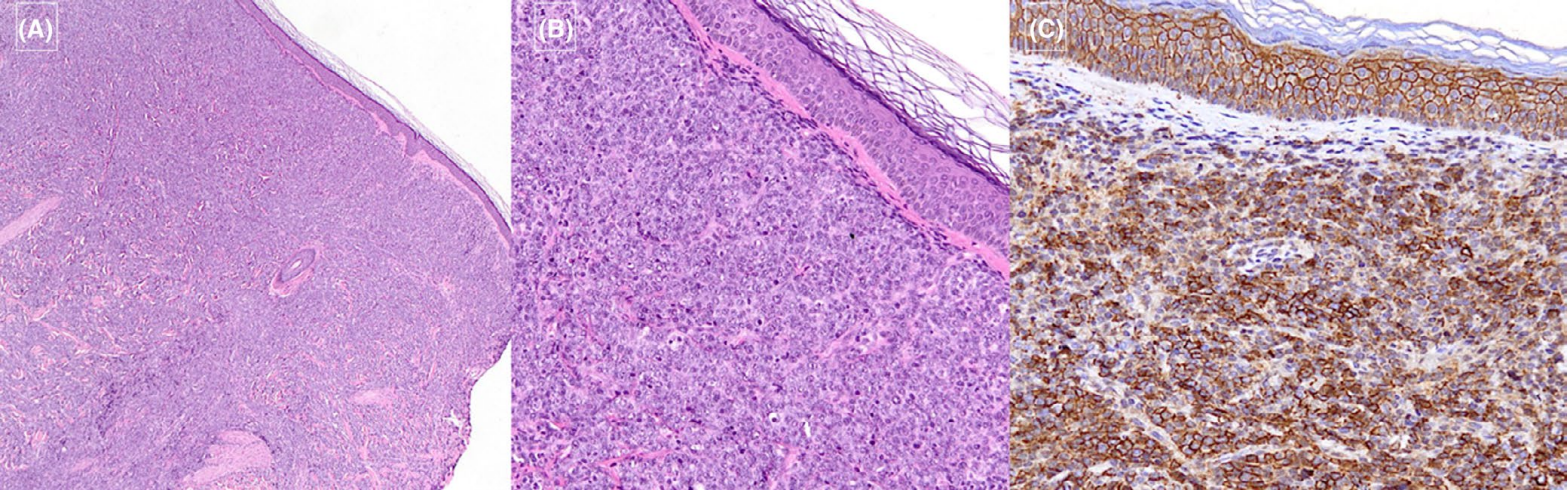
(A) Dense infiltrate of neoplastic plasma cells throughout the dermis, sparing the epidermis (H&E 2×). (B) Higher magnification showing pleomorphism and high mitotic activity (H&E 10×). (C) Immunohistochemistry staining CD138 turned out to be positive (10×).

Dermoscopic examination revealed superficial scales overlying a pinkish background, along with widespread white‐yellowish dots of various sizes (Figure 1B). A 4‐mm biopsy from the largest lesion showed a diffuse dermal non‐epidermotropic infiltrate, consisting of large/sized atypical elements with plasma cell differentiation, nuclear pleomorphism, and high mitotic activity. A grenz zone of uninvolved superficial dermis was observed. At immunohistochemistry, the neoplastic population showed a diffuse, and intense expression of CD138, negativity of CD3, and CD20 and high proliferation index Ki67 (90% of the cell +), confirming the clinical suspicion of a cutaneous secondary localization of multiple myeloma (Figure 2).

## 3 DISCUSSION

Multiple myeloma (MM) is a hematological disorder characterized by the infiltration and proliferation of malignant monoclonal plasma cells, primarily in the bone marrow, resulting in the production of a monoclonal protein.^1^ Cutaneous involvement in MM is uncommon, occurring in 1%–4% of patients, usually showing bone marrow involvement preceding the cutaneous lesions.^2^ Cutaneous involvement presents as nodules resembling lymphoma or as various dermatological manifestations.^3^ These may include plasmacytomas, secondary amyloidosis, sarcoidosis, or paraneoplastic syndromes. Most extramedullary plasmacytomas appear in the upper airways and gastrointestinal tract, but cutaneous involvement has been seldom described in association with advanced disease. Cutaneous plasmacytoma (CP) generally presents with nodules or multiple plaques of erythematous or purplish color, without specific localization, but with predominance on the trunk.^2^ Differential diagnoses for cutaneous lesions in MM include other nodular lesions presenting with a vascular pattern, such as nonmelanoma skin cancer, that is frequent in patients with chronic immunosuppression, hemangioma or poroma.^3^

In our case, the biopsy was crucial to exclude recurrence of previous carcinomas and to differentiate CP from other skin lesions or paraneoplastic processes associated with MM or its complications. Despite therapeutic interventions, the prognosis for cutaneous MM remains unfavorable. Following the histological examination, a FDG PET‐CT revealed extramedullary extension of myeloma involving the left pelvis, retroperitoneum, and left paravertebral muscle, along with uptake in the two skin nodules. Blood tests indicated elevated erythrocyte sedimentation rate, C‐reactive protein, creatinine, and lactate dehydrogenase, with decreased total proteins, IgG, IgM, and a significant imbalance in the serum free Kappa/Lambda ratio, consistent with the diagnosis of MM. The patient was referred to hematologists, starting an alternative therapeutic cycle with belantamab mafodotin. She died after the third cycle of therapy.

## AUTHOR CONTRIBUTIONS

**L. Rapparini:** Conceptualization; data curation; investigation; writing – original draft. **A. Pileri:** Investigation; methodology; supervision; validation; visualization. **S. Robuffo:** Investigation; supervision; validation; visualization. **C. Agostinelli:** Supervision; validation; visualization. **Michelangelo La Placa:** Conceptualization; data curation; formal analysis; investigation; methodology; writing – review and editing.

## FUNDING INFORMATION

None.

## CONFLICT OF INTEREST STATEMENT

The authors declare no conflicts of interest.

## CONSENT

I confirm that written patient consent has been signed and collected in accordance with the journal's patient consent policy, and that I will retain the original written consent form and provide it to the Publisher if requested.

## Data Availability

Data are available upon request from the authors.
